# Structures of *Pseudomonas aeruginosa* β-ketoacyl-(acyl-carrier-protein) synthase II (FabF) and a C164Q mutant provide templates for antibacterial drug discovery and identify a buried potassium ion and a ligand-binding site that is an artefact of the crystal form

**DOI:** 10.1107/S2053230X15010614

**Published:** 2015-07-28

**Authors:** Bernhard Baum, Laura S. M. Lecker, Martin Zoltner, Elmar Jaenicke, Robert Schnell, William N. Hunter, Ruth Brenk

**Affiliations:** a Institut für Pharmazie und Biochemie, Johannes Gutenberg-Universität, Staudinger Weg 5, 55128 Mainz, Germany; b Division of Biological Chemistry and Drug Discovery, College of Life Sciences, University of Dundee, Dundee DD1 4EH, Scotland; c Institut für Molekulare Biophysik, Johannes Gutenberg-Universität, Jakob Welder Weg 26, 55128 Mainz, Germany; d Department of Medical Biochemistry and Biophysics, Karolinska Institutet, 17 177 Stockholm, Sweden

**Keywords:** fatty-acid biosynthesis, FabF, Gram-negative bacteria, potassium binding, *Pseudomonas aeruginosa*, structure-based drug discovery

## Abstract

Bacterial infections remain a serious health concern, in particular causing life-threatening infections of hospitalized and immunocompromised patients. The situation is exacerbated by the rise in antibacterial drug resistance, and new treatments are urgently sought. In this endeavour, accurate structures of molecular targets can support early-stage drug discovery. Here, crystal structures, in three distinct forms, of recombinant *Pseudomonas aeruginosa* β-ketoacyl-(acyl-carrier-protein) synthase II (FabF) are presented. This enzyme, which is involved in fatty-acid biosynthesis, has been validated by genetic and chemical means as an antibiotic target in Gram-positive bacteria and represents a potential target in Gram-negative bacteria. The structures of apo FabF, of a C164Q mutant in which the binding site is altered to resemble the substrate-bound state and of a complex with 3-(benzoylamino)-2-hydroxybenzoic acid are reported. This compound mimics aspects of a known natural product inhibitor, platensimycin, and surprisingly was observed binding outside the active site, interacting with a symmetry-related molecule. An unusual feature is a completely buried potassium-binding site that was identified in all three structures. Comparisons suggest that this may represent a conserved structural feature of FabF relevant to fold stability. The new structures provide templates for structure-based ligand design and, together with the protocols and reagents, may underpin a target-based drug-discovery project for urgently needed antibacterials.

## Abstract 2

Three crystal structures of recombinant *P. aeruginosa* FabF are reported: the apoenzyme, an active-site mutant and a complex with a fragment of a natural product inhibitor. The characterization provides reagents and new information to support antibacterial drug discovery.

## 1. Introduction   

*Pseudomonas aeruginosa* is a Gram-negative bacterium that is responsible for a significant level of hospital-acquired infections, particularly in burn victims, immunocompromised and cystic fibrosis patients (Kerr & Snelling, 2009 ▸; Ratjen & Döring, 2003 ▸). There is an urgent need for the development of new antibiotics that tackle this and related drug-resistant Gram-negative bacteria (Payne *et al.*, 2007 ▸; Shlaes, 2003 ▸). One way forward involves a structure-based approach, in particular by exploiting accurate chemical information provided by the crystallographic analyses of carefully considered molecular targets (Hunter, 2009 ▸). A potential target for antibiotic drug discovery is β-ketoacyl-(acyl-carrier-protein) synthase II (FabF), which is one of the essential elongation condensing enzymes of fatty-acid biosynthesis (Wang *et al.*, 2006 ▸), a highly conserved metabolic pathway among key microbial pathogens. During fatty-acid elongation the acyl-carrier protein (ACP) substrate transfers a fatty acid to the S atom of the catalytic cysteine of FabF. The malonyl-ACP substrate then interacts with the acyl-enzyme intermediate and forms a β-ketoacyl-ACP product. FabF requires a catalytic triad (Zhang *et al.*, 2006 ▸), which in the *P.* aeruginosa enzyme (*Pa*FabF) comprises Cys164, His304 and His341.

**Figure 1:**
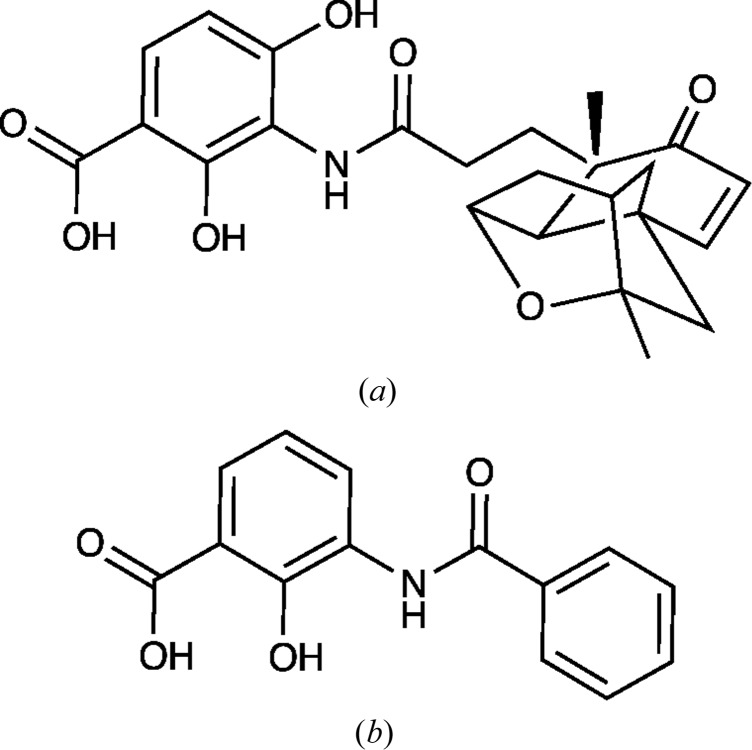
Chemical structures of (*a*) platensimycin and (*b*) 3-(benzoylamino)-2-hydroxybenzoic acid (compound **1**).

FabF has been validated in Gram-positive bacteria as the target for the natural products platensimycin (Fig. 1 ▸; Wang *et al.*, 2006 ▸) and fasamycin A and B (Feng *et al.*, 2012 ▸). Platensimycin, although inactive against wild-type Gram-negative bacteria, is active against efflux-negative *Escherichia coli* (Wang *et al.*, 2006 ▸). Similarly, the fasamycins are active against membrane-permeabilized *Escherichia coli* but not the unaltered strain (Feng *et al.*, 2012 ▸). These observations suggest that the absence of activity against Gram-negative bacteria of these FabF inhibitors is owing to their inability to access their target. This issue might potentially be overcome by developing chemical matter based on different scaffolds which are more easily taken up by Gram-negative bacteria and that might avoid efflux mechanisms.

We now report the preparation and use of an efficient recombinant protein-production system and purification and crystallization protocols for the enzyme from *P. aeruginosa* (*Pa*FabF; UniProt code G3XDA2) and for an active-site C164Q mutant. The mutant is believed to mimic the substrate-bound state of FabF (Wang *et al.*, 2006 ▸). Crystal structures of both the apoenzyme and mutant are reported. These provide accurate templates to support structure-based approaches to search out molecules with the right properties to tackle Gram-negative bacteria. In *Pa*FabF we noted a buried, highly ordered potassium-binding site that might represent a conserved feature of FabF that is important for fold stability. Comparison with the previously known structure of the enzyme from *E. coli* (*Ec*FabF; PDB entry 2gfw; Wang *et al.*, 2006 ▸) revealed the highly conserved character of the overall structure, the catalytic centre and the monovalent cation-binding site. Attempts to co-crystallize *Pa*FabF and the mutant with commercially available small-molecule fragments of platensimycin resulted in the structure of the complex with compound **1** [3-(benzoylamino)-2-hydroxybenzoic acid; Fig. 1 ▸]. Surprisingly, this compound did not bind in the active site but rather occupies a site between two symmetry-related molecules.

## 2. Methods   

### 2.1. Recombinant protein production and purification   

*Pa*FabF was obtained following a similar strategy to one described previously (Moynie *et al.*, 2013 ▸). The gene was cloned into the pNIC28-Bsa4 vector (GenBank ID EF198106; Stols *et al.*, 2002 ▸) encoding a tobacco etch virus (TEV) cleavage site and an N-terminal hexahistidine tag. The new plasmid was heat-shock transformed into BL21(DE3)pLysS competent *E. coli* cells (Novagen), which were cultured in 1 l autoinduction medium (MgSO_4_/NPS-5052) supplemented with kanamycin (50 mg ml^−1^) and chloramphenicol (25 mg ml^−1^) for 48 h at 293 K. The cells were harvested by centrifugation (30 min, 3500*g*, 277 K), resuspended in lysis buffer [25 m*M* Tris–HCl pH 8.5, 500 m*M* NaCl, 20 m*M* imidazole, one tablet of protease-inhibitor EDTA-free cocktail (Roche), 0.1 mg DNase (Sigma)] and lysed by mechanical disruption using a French pressure-cell press at 110 MPa. Centrifugation of the sample (30 min, 277 K, 40 000*g*) was followed by passage through 0.2 µm filters to remove debris. The protein was then purified using an Ni^2+^ Sepharose High Performance HisTrap HP 5 ml column (GE Healthcare) with an increasing imidazole gradient to 500 m*M* for elution. The fractions containing *Pa*FabF were pooled and His-tagged TEV protease was added to remove the affinity tag. The mixture was dialyzed with buffer (25 m*M* Tris–HCl pH 7.5, 150 m*M* NaCl) overnight at 277 K and the cleaved protein was purified by passage through an Ni^2+^-charged HisTrap column. Size-exclusion chromatography was then performed on a Superdex 200 HiLoad 26/60 prep-grade column (GE Healthcare) with equilibration buffer (25 m*M* Tris–HCl pH 7.5, 150 m*M* NaCl). The column had previously been calibrated with molecular-mass standards (thyroglobulin, 670 kDa; γ-globulin, 158 kDa; serum albumin, 67 kDa; ovalbumin; 44 kDa, myoglobin, 17 kDa; vitamin B_12_, 1 kDa). A high level of purity was confirmed by SDS–PAGE (Mini-PROTEAN TGX Stain-Free Precast Gel; Bio-Rad) and the recovery of *Pa*FabF was determined using a NanoVue spectrophotometer (GE Healthcare).

### 2.2. Protein production and purification of the *Pa*FabF C164Q mutant   

*In vitro* site-directed mutagenesis using the Agilent QuikChange kit was carried out using the *Pa*FabF gene carrying the pNic28-Bsa4 vector to mutate Cys164 to Gln164. The PCR product generated using the forward primer 5′-GCCCTCACCACCGCC**CAG**ACCACCGGTACCCAC-3′ and the reverse primer 5′-GTGGGTACCGGTGGT**CTG**GGCGGTGGTGAGGGC-3′ was treated with the DpnI restriction enzyme (Biolabs) to digest the methylated template plasmid. The restriction sites are shown in bold. The *Pa*FabF-C164Q encoding plasmid was amplified by transforming *E. coli* XL1-Blue supercompetent cells (Novagen). Plasmid DNA was extracted (QIAprep Spin Miniprep Kit, Qiagen) and verified by DNA sequencing (DNA Sequencing Unit, University of Dundee). Overexpression and protein purification were performed as described for wild-type *Pa*FabF but using phosphate buffer [50 m*M* Na_2_HPO_4_ pH 7.8, 300 m*M* NaCl, 10%(*v*/*v*) glycerol, 0.5 m*M* DTT]. This was necessary to avoid precipitation of the mutant protein.

### 2.3. Crystallization and X-ray data collection   

Crystallization screens were performed with a Phoenix liquid-handling system (Art Robbins Instruments, Rigaku) using sitting-drop vapour-diffusion conditions in 96-well plates and commercial screens from Hampton Research and Qiagen. Optimization led to planar crystals (250 × 100 × 10 µm) using a reservoir consisting of 0.2 *M* MgCl_2_, 0.1 *M* Tris–HCl pH 7.0, 10%(*w*/*v*) PEG 8000 and a protein solution consisting of 20 mg ml^−1^ *Pa*FabF in 25 m*M* Tris–HCl pH 7.5, 150 m*M* NaCl. Plate-shaped crystals (100 × 100 × 5 µm) of *Pa*FabF-C164Q were obtained at 20 mg ml^−1^ [in 50 m*M* Na_2_HPO_4_ pH 7.8, 150 m*M* NaCl, 10%(*v*/*v*) glycerol, 0.5 m*M* DTT] with a reservoir consisting of 0.2 *M* NH_4_HCO_2_, 25%(*w*/*v*) PEG 3350. For both the wild-type and the mutant *Pa*FabF crystals, 1 µl protein solution and 1 µl reservoir solution were equilibrated against 60 µl reservoir solution in sitting drops at 293 K in MRC crystallization plates (catalogue No. MD11-00-10, Molecular Dimensions). For the structure of the complex with compound **1**, 1 µl wild-type protein (20 mg ml^−1^ in 25 m*M* Tris–HCl pH 7.5), 0.5 µl of a 10 m*M* solution of compound **1** in DMSO and 0.5 µl reservoir solution were equilibrated against 60 µl reservoir solution [0.2 *M* MgCl_2_, 0.1 *M* Tris–HCl pH 7.0, 10%(*w*/*v*) PEG 8000]. Attempts to co-crystallize compound **1** with the mutant protein were unsuccessful. Compound **1** was ordered from Vitas-M Laboratory Ltd (code STK685369) and its identity and purity (>99%) were verified by LC-MS (data not shown).

Crystals were transferred to a cryoprotectant based on the reservoir adjusted to contain 25%(*v*/*v*) glycerol prior to flash-cooling in liquid nitrogen. X-ray diffraction data for the apo and mutant structures were collected from single crystals with a MicroMax-007 HF X-ray generator equipped with a Saturn 944 HG CCD detector (Rigaku). The data for the complex structure were collected from a single crystal on a Bruker AXS Microstar-H generator with a MAR Scanner 345 mm image-plate detector. In each case crystals were maintained at 100 K and the X-ray wavelength was 1.5418 Å (Cu *K*α). Data were processed using *XDS* (Kabsch, 2010 ▸) and *SCALA* (Evans, 2006 ▸).

### 2.4. Structure solution and refinement   

The structures were solved by molecular replacement using *Phaser* (McCoy, 2007 ▸). The apo *Pa*FabF form was first determined using the homologous monomer (chain *A*) of *Ec*FabF (PDB entry 2gfw), with 67% sequence identity, as a search model. The structure of *Pa*FabF was refined and subsequently used as the starting model for analysis of the other two structures. Refinements involved least-squares calculations performed in *REFMAC*5 (Murshudov *et al.*, 2011 ▸) interspersed with inspection of electron-density and difference density maps and model manipulation using *Coot* (Emsley & Cowtan, 2004 ▸). During the course of the refinements, water molecules and side-chain conformers were included. The model geometry was assessed using *MolProbity* (Chen *et al.*, 2010 ▸) and the PDB validation tools. The properties of the dimer interface were investigated with *PISA* (Krissinel & Henrick, 2007 ▸). The crystallographic data and refinement statistics are listed in Table 1 ▸. The figures were generated with *PyMOL* v.1.5.0.4 (Schrödinger).

## 3. Results and discussion   

### 3.1. Structure of apo *Pa*FabF   

**Figure 2:**
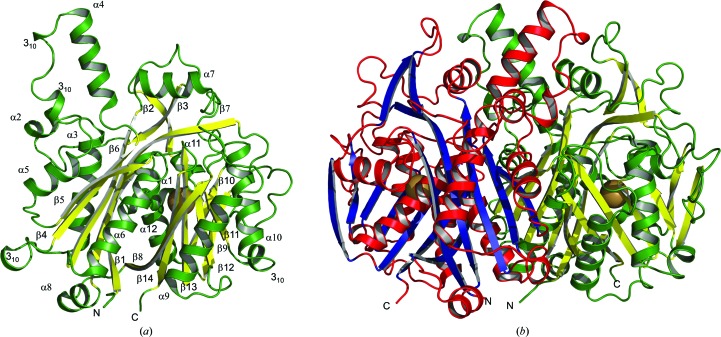
Overview of the apo crystal structure. (*a*) Secondary-structure elements of the *Pa*FabF subunit. Helices are displayed in green and strands in yellow. Secondary-structure elements and the N-terminus and C-terminus are labelled. The buried potassium ion is shown as a brown sphere. (*b*) Dimer of *Pa*FabF. The monomers are displayed in green/yellow and red/blue colour combinations. The potassium ions are shown as brown spheres and the C- and N-termini are labelled.

An efficient recombinant *Pa*FabF production system has been established that produces in excess of 10 mg of pure enzyme per litre of bacterial culture. The apoenzyme crystallized in space group *P*2_1_ with two molecules in the asymmetric unit and the structure was determined to 1.73 Å resolution (Table 1 ▸, Fig. 2 ▸). Size-exclusion chromatography, which was used during purification, consistently indicated a single species with a molecular mass of approximately 55 kDa. This value lies between the theoretical values for a monomer (43 kDa) and a dimer (86 kDa). Analysis of the crystal structure using *PISA* (Krissinel & Henrick, 2007 ▸) suggested that the dimer present in the asymmetric unit is stable in solution. The accessible surface area of a subunit is estimated to be 15 460 Å^2^ and the area occluded by dimer formation is about 2840 Å^2^. The dimer interface is therefore formed using approximately 20% of the total subunit surface and the solvation free-energy gain is estimated to be −221 kJ mol^−1^, suggesting a stable entity. The presence of a *Pa*FabF dimer is also consistent with previous work on the *E. coli* enzyme (Edwards *et al.*, 1997 ▸).

The *Pa*FabF subunit consists of 14 β-sheets, 12 α-helices and four 3_10_-helices (Fig. 2 ▸). The β-strands can be divided into two sets of five. β4, β5 and β6 are parallel and β1 and β7 are antiparallel and form the first unit, whereas the parallel β9, β10 and β13 and the antiparallel β8 and β14 form the second unit. These two sections enclose the two helices α6 and α12. The two sets of helices α3, α5 and α9, α10 are packed against the domain. Accordingly, the monomer exhibits a globular five-layered structure, α–β–α–β–α. The overall fold and architecture of the active site is highly conserved in orthologous structures, for example the *E. coli* enzyme (Huang *et al.*, 1998 ▸).

The *Pa*FabF dimer displays approximate dimensions of 60 × 50 × 40 Å (Fig. 2 ▸). A large dimer interface area, with significant hydrophobic properties, maintains the quaternary structure. Hydrophobic interactions are mainly observed through the self-association of residues in α7, α5 and α4, the two 3_10_-helices and β5. The β-sheets of each subunit run antiparallel to each other, which thus forms a continuous sheet through the interface of the two monomers. The loop bordered by β4 and α5, containing α4 and two 3_10_-helices, is in close interaction with the related loop in the other monomer. Helix α5 is also in contact with the C-terminal end of β8 and β13 of the second monomer.

### 3.2. The active site and the C164Q mutant   

**Figure 3:**
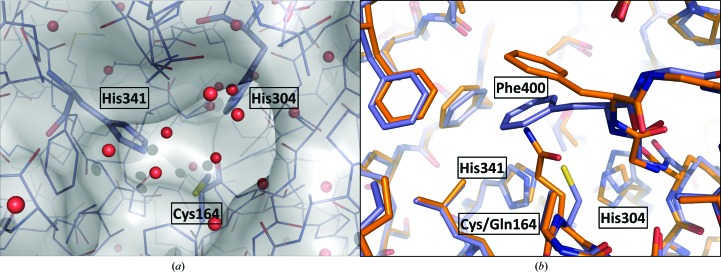
The active site. (*a*) A view into the narrow active-site cleft of the wild-type enzyme. The van der Waals radii are depicted as a grey semi-transparent surface. Residues are shown as sticks coloured by atomic position (purple, C; red, O; blue, N; yellow, S). Red spheres are waters. Three key catalytic residues are labelled. (*b*) Superposition of the wild-type and C164Q mutant active sites. The same colour scheme for atoms is used as in (*a*) except that the C atoms of the mutant are in orange. As a result of the C164Q mutation, Phe400 rotates out of the binding pocket.

There is one active site in each subunit. This is a narrow cleft in which the three catalytic residues are placed (Fig. 3 ▸). To further investigate the active site, a *Pa*FabF-C164Q mutant was generated that mimics the acyl-enzyme intermediate, thereby forming a competent state to bind the inhibitor platensimycin (Wang *et al.*, 2006 ▸). The *Pa*FabF-C164Q mutant crystallized in a different space group (*C*222_1_; Table 1 ▸) compared with the wild-type structure and the structure was determined to 2.40 Å resolution. A dimer constitutes the asymmetric unit and the structure is essentially identical to that of the wild type, with an r.m.s.d. of 0.24 Å for the overlay of all C^α^ atoms. The binding-site residues are also well aligned, although we note that Phe400 is rotated away from the catalytic centre owing to an extension in the length of residue 164, where glutamine replaces cysteine (Fig. 3 ▸). This results in a binding pocket that is slightly more open and Phe400 can then participate in hydrophobic interactions with a symmetry-related molecule. Such a change may contribute to the different crystal form that is observed for the mutant.

### 3.3. A potassium ion binding site   

**Figure 4:**
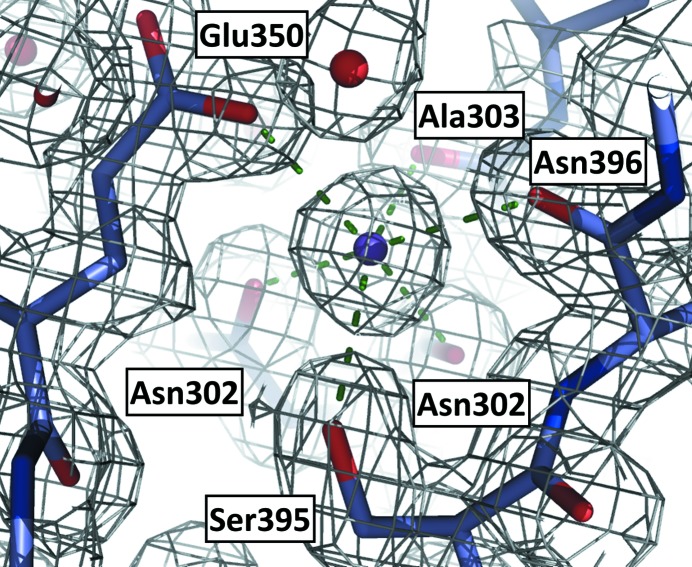
Coordination of the buried potassium ion in *Pa*FabF. The grey chicken wire is 2*F*_o_ − *F*_c_ density. Green dashed lines represent the coordination links between the cation and functional groups on the protein.

A striking and unusual observation is the identification of a buried cation approximately 8 Å from the catalytic Cys164 in all three structures. Care was taken in the assignment of this ion based on correlation of difference density (*F*_o_ − *F*_c_) σ levels with the number of electrons (Fyfe *et al.*, 2009 ▸; Rimsa *et al.*, 2011 ▸). The exclusion of all eight S atoms in *Pa*FabF produced positive peaks in the difference density map with an average height of 18.6σ. At the position of the ion an average peak height of 21.9σ was obtained. This, together with the coordination geometry, is consistent with the assignment of a potassium ion. In all three structures the satisfactory refinement of potassium ions with full occupancy was noted and resulted in the same octahedral environment for the ion. The octahedral coordination involves O atoms from five different residues (Fig. 4 ▸). Asn302 coordinates in a bidentate mode to the potassium ion using the side-chain and main-chain O atoms. Other coordinating O atoms are from the side chains of Glu350 and Ser395 and the main chains of Ala303 and Asn396. The distances between the ion and the coordinating atoms are within the predicted 2.59–2.97 Å distances noted for potassium ion binding (Harding, 2002 ▸). The side-chain atoms and the potassium ion show similar *B* factors of approximately 13 Å^2^. No potassium was added to the buffers that were used for purification or to the crystallization reagents; therefore, we conclude that this group I ion was acquired during production in the heterologous expression system.

The fact that the ion is completely buried and has a coordination sphere encompassing residues in different elements of secondary structure suggests a contributing role whereby this polypeptide folds into a catalytically competent entity. If this were true it might be expected that FabF orthologues could show a similar feature and, indeed, comparison with other FabF structures shows that this might be the case. The two asparagine residues, the alanine and glutamate are highly conserved in FabF sequences and structures. The serine, whilst well maintained, is conservatively substituted by cysteine or threonine. The 1.9 Å resolution structure of *Ec*FabF (PDB entry 3i8p; Singh *et al.*, 2009 ▸) has a buried water assigned at this site, with similar geometry to that we observe except that Cys395 replaces the serine. The *B*-factor value of about 6 Å^2^ for this water molecule is one third of that of the coordinating groups, which are amongst the most ordered in *Ec*FabF, and the difference density map reveals a positive peak of 6σ at the water position. We judge it likely that this is actually a cation and not a water and is likely to be potassium. Other FabF structures at lower resolution all have highly ordered water molecules assigned at this position with at least fivefold coordination and a geometry that would be consistent with cation binding. This is exemplified by the structures of the enzymes from *Vibrio cholerae* (PDB entry 4jrm; Center for Structural Genomics of Infectious Diseases, unpublished work) and *Bacillus subtilis* (PDB entry 4ls8; Trajtenberg *et al.*, 2014 ▸). Here also, the only amino-acid difference at the potassium ion-binding site involves the replacement of the serine with Cys397 in the *V. cholerae* enzyme. A polyketide synthase fold relative (PDB entry 1tqy; Keatinge-Clay *et al.*, 2004 ▸) displays a similar buried cation-binding site where a magnesium ion is assigned. Ser395 and Asn396 of *Pa*FabF correspond to Thr400 and Val401 in this related structure. In the latter case it is the main chain that interacts with the cation, so the nature of the side chain is less important. This buried ion has escaped comment but we judge it to be supportive of our conclusion that such a site might be particularly relevant to the stability of this enzyme fold.

### 3.4. Complex with compound **1**   

**Figure 5:**
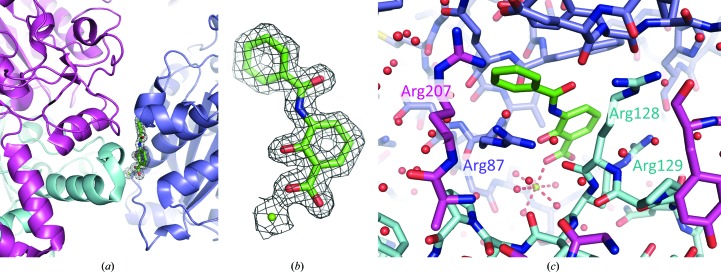
Binding of compound **1**. (*a*) Three symmetry-related subunits shown as different coloured ribbons (light blue, magenta and cyan) around the binding site of compound **1** (shown as sticks with C atoms in green). (*b*) The OMIT difference *F*_o_ − *F*_c_ density (green chicken wire, 1σ level) for compound **1** and a magnesium ion (green sphere). (*b*) View of the binding site of compound **1**, with amino acids shown as sticks and the same colour code as in (*a*). Selected residues are labelled. The carboxylic group forms interactions with the magnesium ion and surrounding water molecules.

Compounds representing fragments of platensimycin were sought to investigate the molecular-recognition properties of the substrate-binding site. To this end, the ChemSpider database (http://www.chemspider.com) was used to identify compounds containing an aromatic substructure with a similar substitution pattern as in platensimycin. After visual inspection, 20 compounds were purchased for further evaluation. In the absence of an established binding or inhibition assay, we attempted to use crystallographic methods to investigate binding. Soaking of pre-formed apoenzyme crystals in solutions of potential ligands led to degradation of the crystalline order or to no electron density for the ligands. The compounds were also subjected to co-crystallization trials with the wild type and the *Pa*FabF-C164Q mutant and gave only a single success. A new crystal form (*Pa*FabF–compound **1**) in space group *P*4_3_2_1_2 resulted and the structure was determined to 1.67 Å resolution (Table 1 ▸). A single subunit is present in the asymmetric unit and a crystallographic twofold axis of symmetry generates the dimer observed in all other FabF structures. Unexpectedly, the potential ligand did not occupy the active site, but difference density was clearly visible on the surface of the enzyme near two symmetry-related molecules (Fig. 5 ▸). The binding might be considered as that of an ion pair, since Mg^2+^, coordinated by a carboxylic O atom of compound **1**, in addition to numerous solvent-mediated links, helps to stabilize the complex and this crystal form (Fig. 5 ▸). In addition, hydrophobic interactions of compound **1** contribute to the well ordered mode of binding. Adjacent to the divalent cation-binding site, the hydroxybenzoic acid moiety makes van der Waals interactions with Phe83, Val86 and Val151 on one side of the ring and with Arg128 and Arg129 of a symmetry mate on the other. Nearby, but not in direct interaction, is another symmetry-related subunit. The benzoylamino segment is stacked between Tyr62 and Arg87 from one subunit. As the binding site is located at a crystallographic interface it appears that it is a crystallographic artefact, and binding of the compound in aqueous solution cannot be concluded from this crystal structure.

Compound **1** and platensimycin share a common substructure, but we note that we are aware of no evidence which indicates that the former has any inhibitory activity against FabF. Both compounds contain a 3-amino-2-hydroxybenzoic acid moiety, albeit compound **1** lacks a hydroxyl group in the *para* position to the carboxylate. The structure of the *Ec*FabF-C164Q mutant in complex with platensimycin has been determined (Wang *et al.*, 2006 ▸). In this complex, the 2-hydroxy group is not involved in direct interactions and the 4-hydroxy group, which is lacking in compound **1**, forms a hydrogen bond to a water molecule. The carboxylate group of the compound interacts with the catalytic histidine residues and the amide group interacts with a nearby threonine (Wang *et al.*, 2006 ▸). These residues are all strictly conserved. However, access to the depths of the active site of the *Pa*FabF apoenzyme is restricted by the conformations of Phe230 and Phe400. The side chain of the latter residue is forced to adopt a different conformation when the cysteine is changed to a glutamine. The compound **1**–Mg^2+^ ion pair selectively binds into and perhaps contributes to the formation of a stable binding site on the surface of the enzyme distant from the active site, from which it is likely to be occluded by steric hindrance.

## 4. Conclusions   

We have developed an efficient recombinant protein-production system and purification protocols for a potentially valuable antibacterial drug target, *Pa*FabF. Three structures in distinct crystal forms are reported. Our study provides reagents, templates and high-resolution structural information that can inform target-based drug-discovery projects for urgently needed antibacterial substances with a new mode of action.

## Supplementary Material

PDB reference: *Pa*FabF, 4b7v

PDB reference: C164Q mutant, 4jb6

PDB reference: complex with inhibitor fragment, 4jpf

## Figures and Tables

**Table 1:** Data-collection and refinement statistics Values in parentheses are for the highest resolution shell.

Structure	*Pa*FabF	*Pa*FabF-C164Q	*Pa*FabFcompound **1** complex
PDB code	4b7v	4jb6	4jpf
Data collection and processing			
Space group	*P*2_1_	*C*222_1_	*P*4_3_2_1_2
*a*, *b*, *c* ()	71.8, 65.9, 84.8	101.1, 104.6, 141.6	71.6, 71.6, 140.0
, , ()	90.0, 101.9, 90.0	90.0, 90.0, 90.0	90.0, 90.0, 90.0
Matthews coefficient (^3^Da^1^)	2.2	2.2	2.0
Solvent content (%)	45	45	40
Diffraction data			
Resolution range ()	48.851.73 (1.771.73)	15.382.40 (2.462.40)	28.141.67 (1.771.67)
Unique reflections	76169 (4591)	29532 (2014)	43152 (3127)
Multiplicity	4.5	11.5	4.5
*R*_merge_ †	0.04	0.17	0.07
Wilson *B* (^2^)	19.5	31.6	18.5
Completeness (%)	94.2 (59.7)	99.3 (98.5)	99.5 (94.8)
*I*/(*I*)	25.5 (2.3)	16.0 (4.9)	19.8 (1.2)
Refinement			
*R*_work_/*R*_free_ ‡ (%)	18.0/21.1	17.9/27.2	16.9/20.2
No. of reflections for*R*_work_/*R*_free_	72328/3824	27982/1499	40931/2155
Protein residues	822	824	408
Cations	2 K^+^	2 K^+^	1 K^+^, 1 Mg^2+^
Other atoms (compound **1**)			19
Water molecules	645	544	371
R.m.s.d.s			
Bonds ()	0.006	0.013	0.021
Angles ()	1.08	1.60	1.96
Ramachandran plot, residues in (%)			
Favoured regions	97.1	96.5	96.8
Allowed regions	2.9	3.5	2.9
Outlier regions			0.2
Mean *B* factors (^2^)			
Overall	22.2	19.2	17.6
Protein atoms	17.51	18.71	16.40
Water molecules	27.04	24.59	27.81
Ions	16.80	35.54	19.36
Compound **1**			14.50

† *R*_merge_ = 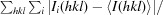 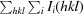, where *I_i_*(*hkl*) is the intensity of the *i*th measurement of reflection *hkl* and *I*(*hkl*) is the mean value of *I_i_*(*hkl*) for all *i* measurements.

‡ *R*_work_ = 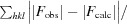 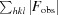, where *F*_obs_ is the observed structure-factor amplitude and *F*_calc_ is the structure-factor amplitude calculated from the model. *R*_free_ is calculated with a subset of data that were excluded from refinement calculations (5%) using the same method as for *R*_merge_.
